# Early-Onset Rheumatic Carditis in a Four-Year-Old: Diagnostic and Immunologic Insights

**DOI:** 10.7759/cureus.102784

**Published:** 2026-02-01

**Authors:** Elma Smajlović, Alma Bolic Alic, Emira Gasal Gvozdenovic

**Affiliations:** 1 Pediatric Cardiology, Zenica Cantonal Hospital, Zenica, BIH; 2 Pediatrics, Zenica Cantonal Hospital, Zenica, BIH; 3 Pediatric Neurology, Zenica Cantonal Hospital, Zenica, BIH

**Keywords:** carditis, revised jones criteria, rheumatic fever, rheumatic heart disease, sydenham’s chorea

## Abstract

Rheumatic fever (RF) is an autoimmune inflammatory disease that occurs several weeks after an episode of pharyngitis caused by Group A β-hemolytic Streptococcus. Rheumatic heart disease (RHD) is a direct consequence of cardiac inflammation and develops through autoimmune mechanisms, including molecular mimicry between streptococcal antigens and host cardiac proteins. Although its global incidence has declined, RF remains endemic in certain regions, and sporadic cases continue to occur in Europe.

We report the case of a three-year-eight-month-old girl presenting with acute behavioral changes and generalized choreiform movements, without preceding infectious symptoms. Elevated anti-streptolysin O titers supported recent streptococcal exposure. Initial echocardiography revealed trivial mitral and aortic regurgitation despite the absence of a murmur; however, serial examinations showed rapid progression to significant regurgitation of both valves. Treatment included oral penicillin, corticosteroids, sodium valproate, and initiation of secondary prophylaxis. Chorea completely resolved within one month. At the 10-month follow-up, aortic regurgitation had resolved, and mitral regurgitation markedly improved.

This case illustrates the diagnostic importance of serial echocardiography in detecting evolving subclinical carditis in patients presenting with Sydenham’s chorea. Although young age is associated with a higher risk of progression, valvular lesions may regress with appropriate therapy and prevention of recurrence.

RF should be considered in choreiform presentations even at very young ages. Early evaluation, repeated echocardiography, and adherence to long-term secondary prophylaxis are essential for preventing disease progression.

## Introduction

Rheumatic fever (RF) is an autoimmune inflammatory disease that occurs several weeks after an episode of pharyngitis caused by Group A β-hemolytic Streptococcus. Rheumatic heart disease (RHD) is a direct consequence of cardiac inflammation. It is believed that RHD results from an autoimmune response to streptococcal antigens through the immunological mechanism of molecular mimicry between streptococcal M protein and host cardiac proteins such as myosin, tropomyosin, vimentin, and laminin [1]. In addition to the heart, inflammation may also involve the brain, joints, and subcutaneous tissues. The severity of the disease and the risk of recurrence are determined by genetic predisposition, pathogen virulence, and immune dysregulation [1].

Globally, the incidence of RF has shown a steady decline, although the disease remains endemic in certain low- and middle-income regions. The incidence of acute RF ranges from less than 1 per 100,000 in middle- to high-income nations to 30-50 per 100,000 in certain endemic areas of sub-Saharan Africa, South Asia, and Oceania [2]. In contrast, the prevalence of the disease has increased due to the widespread use of echocardiography and screening programs, which have identified numerous subclinical cases of carditis. In Europe, RF and RHD are classified as low- to moderate-risk diseases [2].

RF most commonly affects children aged 5-15 years, with a median age of approximately 10 years [3]. The disease is rare in children younger than 5 and in adults older than 30 [3]. Diagnosis is still based primarily on clinical assessment using the revised Jones criteria. Rheumatic carditis remains the most common acquired heart disease in children [4].

## Case presentation

A three-year-eight-month-old girl was admitted to our pediatric department following an acute onset of behavioral changes and abnormal movements of the limbs, body, and head, which resolved during sleep. Symptoms had been present for seven days, with no preceding signs of infection, trauma, or other triggering events. Hoarseness was noted one day prior to admission. One month before symptom onset, she had been treated with an antihistamine for a skin rash. The child had normal early psychomotor development and no known chronic illnesses. Family history revealed that her father was being treated with lamotrigine for epilepsy. On clinical examination, the child was conscious and communicative, with hoarseness, normal heart and lung auscultation, and an unremarkable abdominal examination. Neurological examination revealed choreiform movements. Among a broad panel of laboratory investigations, the only abnormal findings were elevated leukocyte count, C-reactive protein, and antistreptolysin O titers (Table 1).

**Table 1:** Significant laboratory findings Pathological laboratory findings: slightly elevated leukocyte count, elevated C-reactive protein, and elevated antistreptolysin O titer, both at admission and at the peak of symptoms.

Laboratory values	At admission	At the peak of symptoms	Reference values
Leukocyte count	16.67 x 10^9^/L	15.48 x 10^9^/L	5.0-13.0 x 10^9^/L
C-reactive protein (CRP)	10.10 mg/L	56 mg/L	2.87 mg/L
Antistreptolysin O (ASTO) titer	800 IU/ml	800 IU/ml	<200 IU/ml

**Figure 1:**
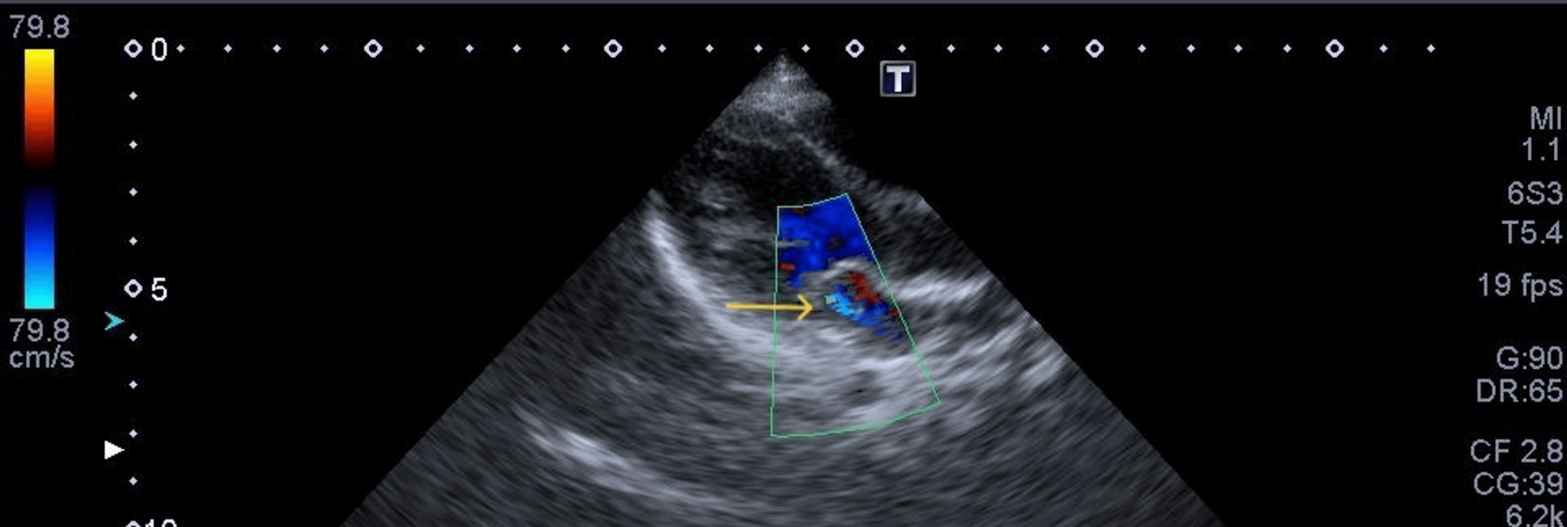
Mitral regurgitation on initial examination Trivial mitral regurgitation on a morphologically normal mitral valve (arrow).

**Figure 2:**
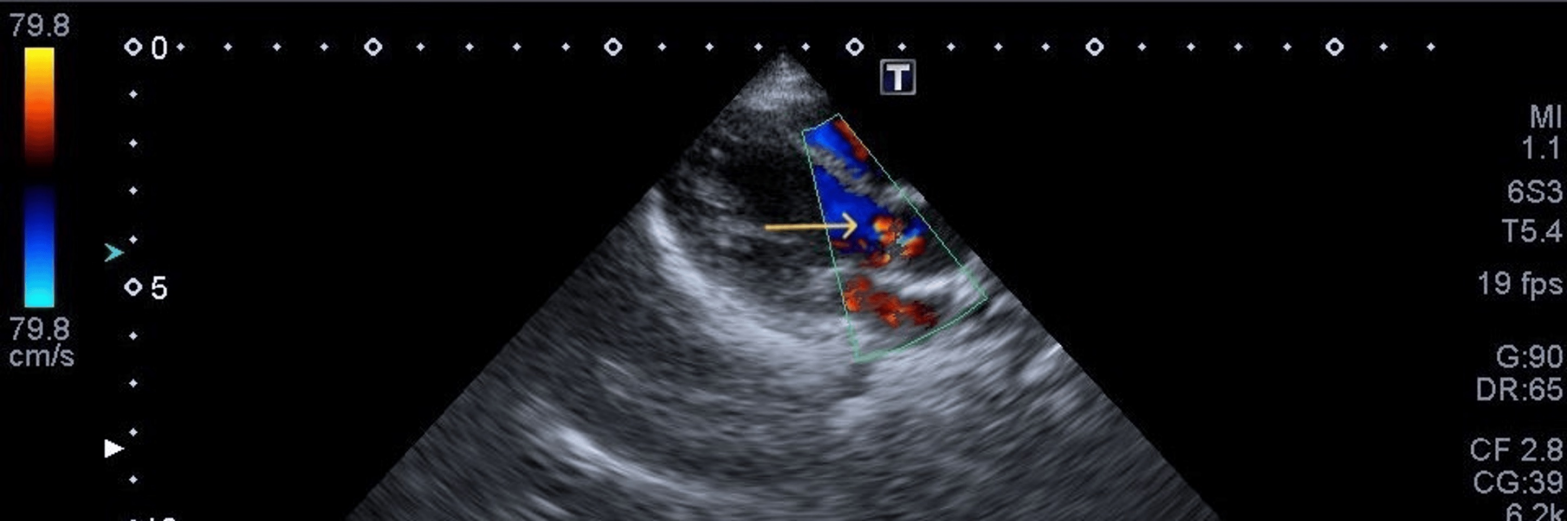
Aortic regurgitation on initial examination Trivial aortic regurgitation on a morphologically normal aortic valve (arrow).

Microbiological testing of stool and urine for bacteria, parasites, and fungi was negative. Throat and nasal swabs for bacterial pathogens were also negative. Enzyme-linked immunosorbent assay (ELISA) testing for Epstein-Barr virus (EBV), cytomegalovirus (CMV), herpes simplex virus (HSV), rubella, and *Toxoplasma gondii* showed negative IgM antibodies; slightly elevated IgG titers for CMV and HSV were considered not clinically significant. Immunological parameters were unremarkable, including negative anti-double-stranded DNA, anti-cyclic citrullinated peptide, antineutrophil cytoplasmic antibody (ANCA) screen, and anti-myeloperoxidase (MPO) antibodies. Neuropediatric evaluation included electroencephalography, which showed no specific abnormalities, and magnetic resonance imaging (MRI) and magnetic resonance angiography (MRA) of the brain, which demonstrated only dilatation of perivascular spaces in the white matter. On the fourth day of hospitalization, the first echocardiographic examination was performed. At that time, the patient had no cardiac symptoms and no audible heart murmur. Electrocardiography showed no abnormalities. Cardiac ultrasound demonstrated normal cardiac function without pericardial effusion, with trivial mitral and aortic regurgitation on morphologically normal valves (Figures 1, 2).

**Figure 3:**
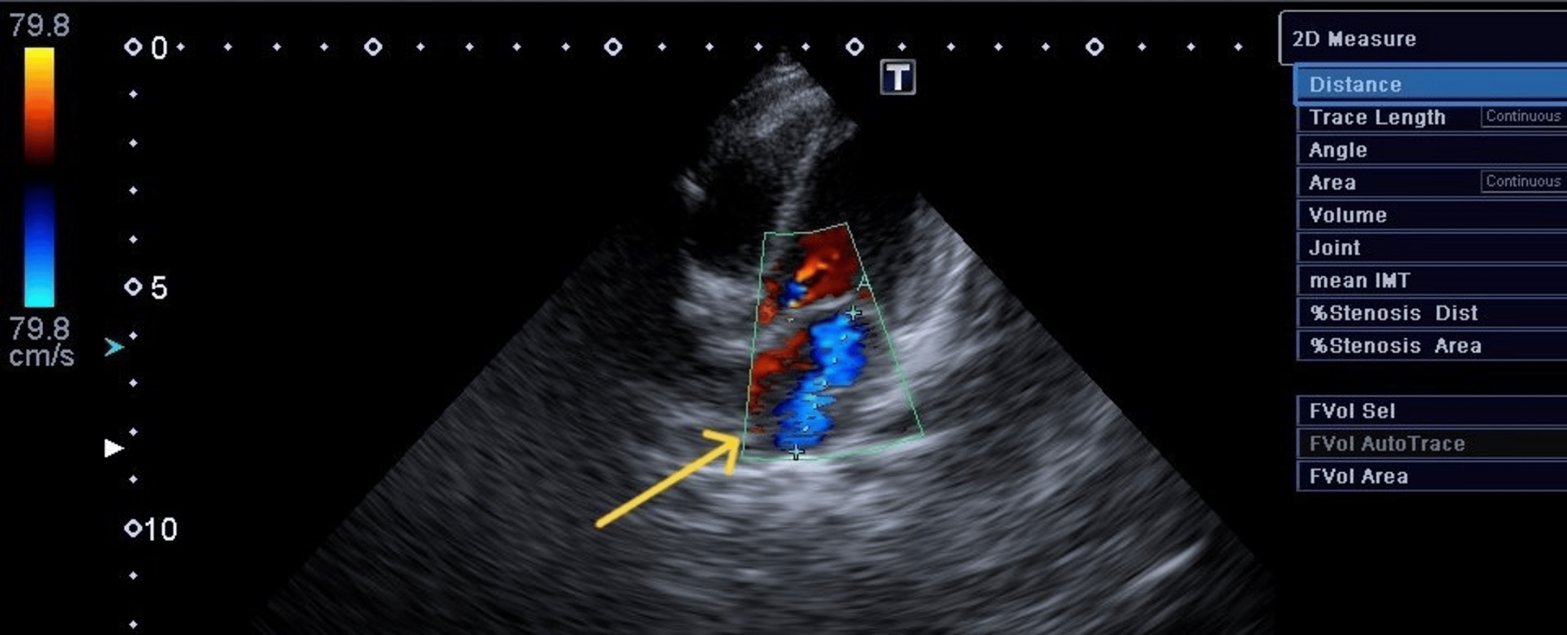
Mitral regurgitation on second examination Mitral regurgitation measuring 30 mm in diameter, with pansystolic jet velocity > 4.0 m/s (arrow).

**Figure 4:**
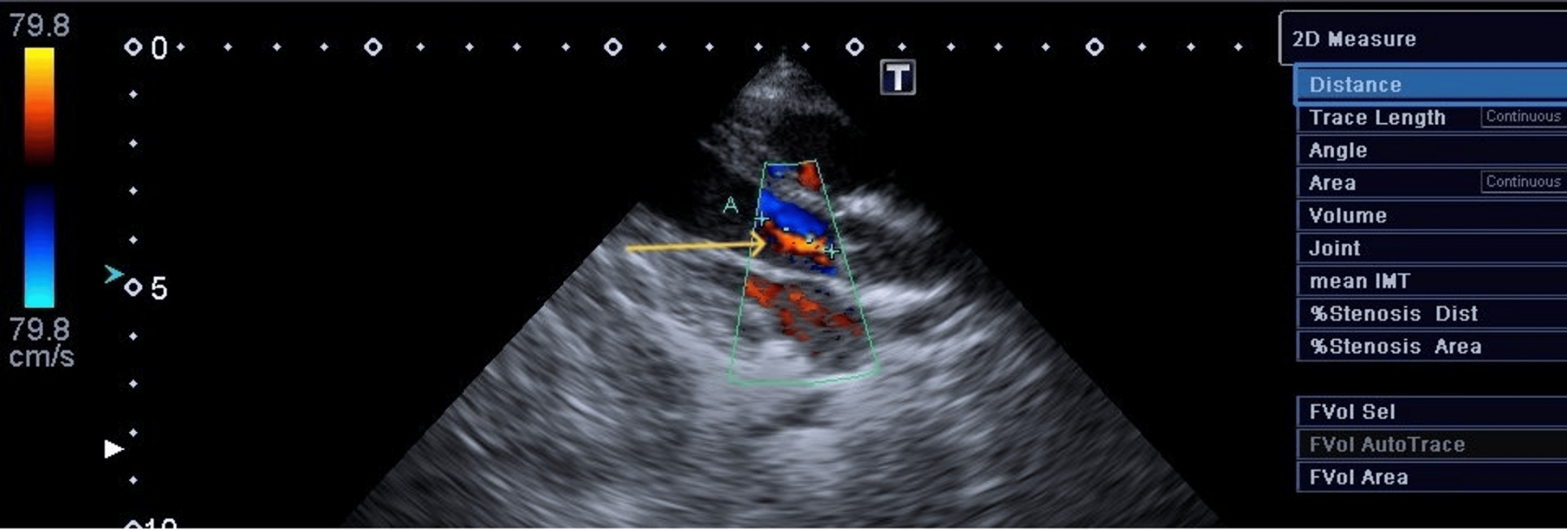
Aortic regurgitation on second examination Aortic regurgitation measuring 16 mm in diameter, with a narrow jet velocity at 2.0 m/s (arrow).

A follow-up echocardiogram performed two days later demonstrated progression of the valvular lesions. Mitral regurgitation measured 30 mm in jet length with a velocity exceeding 4 m/s, while aortic regurgitation measured 1.5 cm in jet length with a velocity of 2.0 m/s (Figures 3, 4).

A grade 2/6 murmur was now auscultated. On the third echocardiographic examination, the murmur had further intensified, accompanied by progression in both the jet diameter and velocity of the mitral regurgitation. Based on these findings, the diagnostic criteria for acute RF with RHD were fulfilled.

A 10-day course of oral penicillin was initiated, followed by prednisone at a dose of 1 mg/kg for two weeks with subsequent gradual tapering. The patient was also treated with sodium valproate for three months and benzodiazepines for symptomatic control, along with secondary antibiotic prophylaxis using benzathine benzylpenicillin.

**Figure 5:**
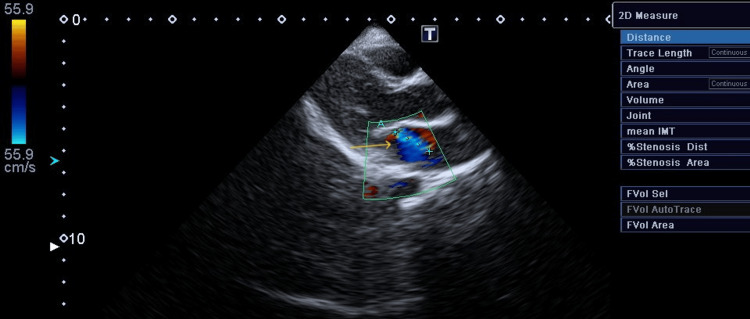
Mitral regurgitation on follow-up examination Echocardiography follow-up demonstrating mitral regurgitation measuring 15 mm in diameter with a velocity of 2.0 m/s (arrow).

At the one-month follow-up, chorea had completely resolved. After two months, the cardiac murmur had decreased in intensity, as had the degree of mitral regurgitation, which measured 1.5 cm with a velocity of 2 m/s, while the aortic valve demonstrated only a trivial regurgitant jet. At 10 months, echocardiographic findings of the mitral valve remained unchanged, and the aortic regurgitation had completely resolved (Figure 5).

**Figure 6:**
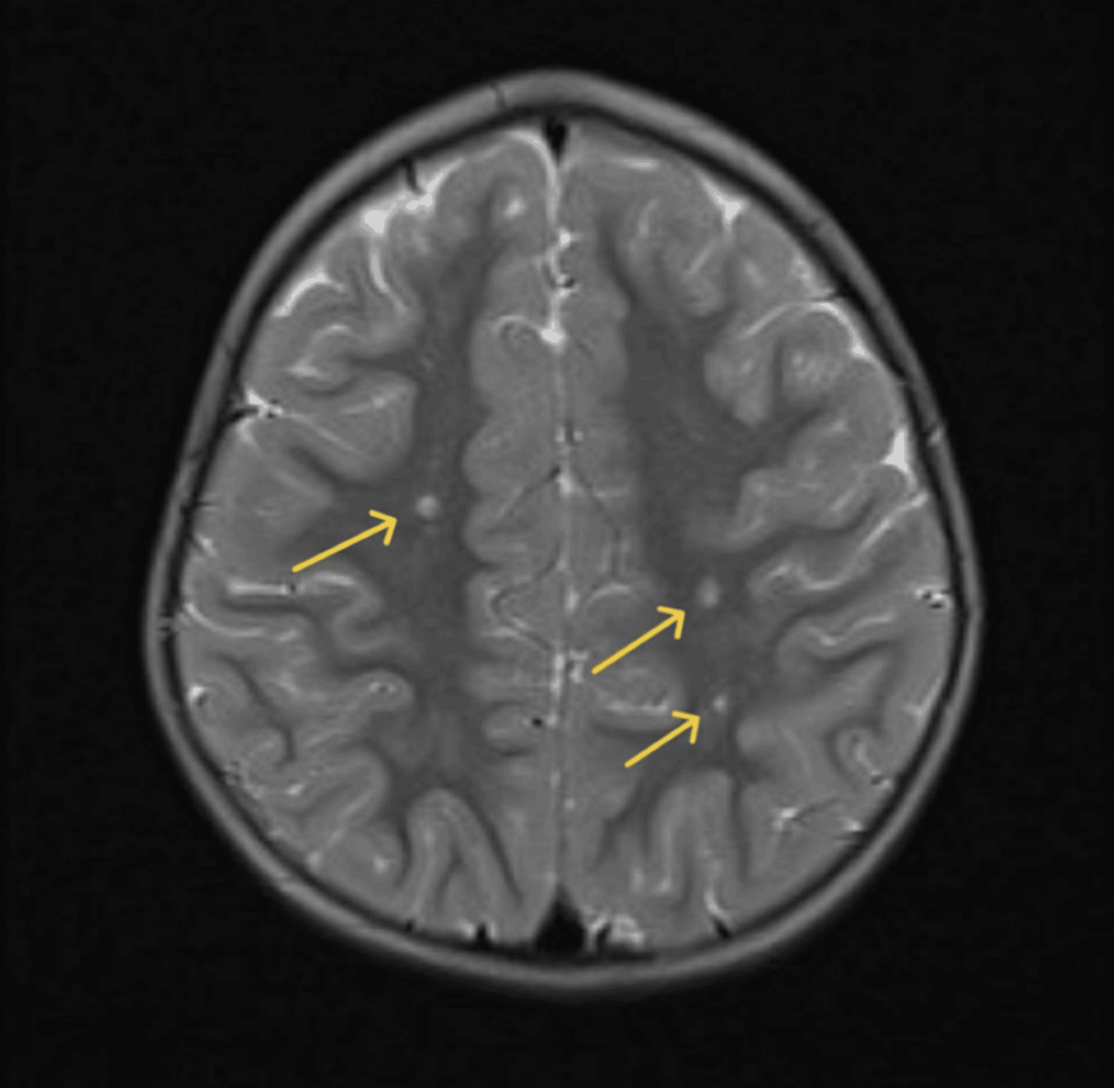
Initial MRI scan: dilated perivascular spaces Axial T2-weighted MRI demonstrating multiple dilated perivascular (Virchow-Robin) spaces within the parasagittal deep and subcortical white matter of bilateral parietal regions. Lesions are well-circumscribed, with CSF-like signal intensity, without surrounding gliosis, mass effect, or cortical involvement (arrows).

Morphologically, the aortic valve showed mildly wrinkled cusps with preserved normal flow. A follow-up brain MRI performed three months later demonstrated persistent dilatation of perivascular spaces within the white matter, consistent with the findings described on the initial MRI study (Figures 5, 6).

**Figure 7:**
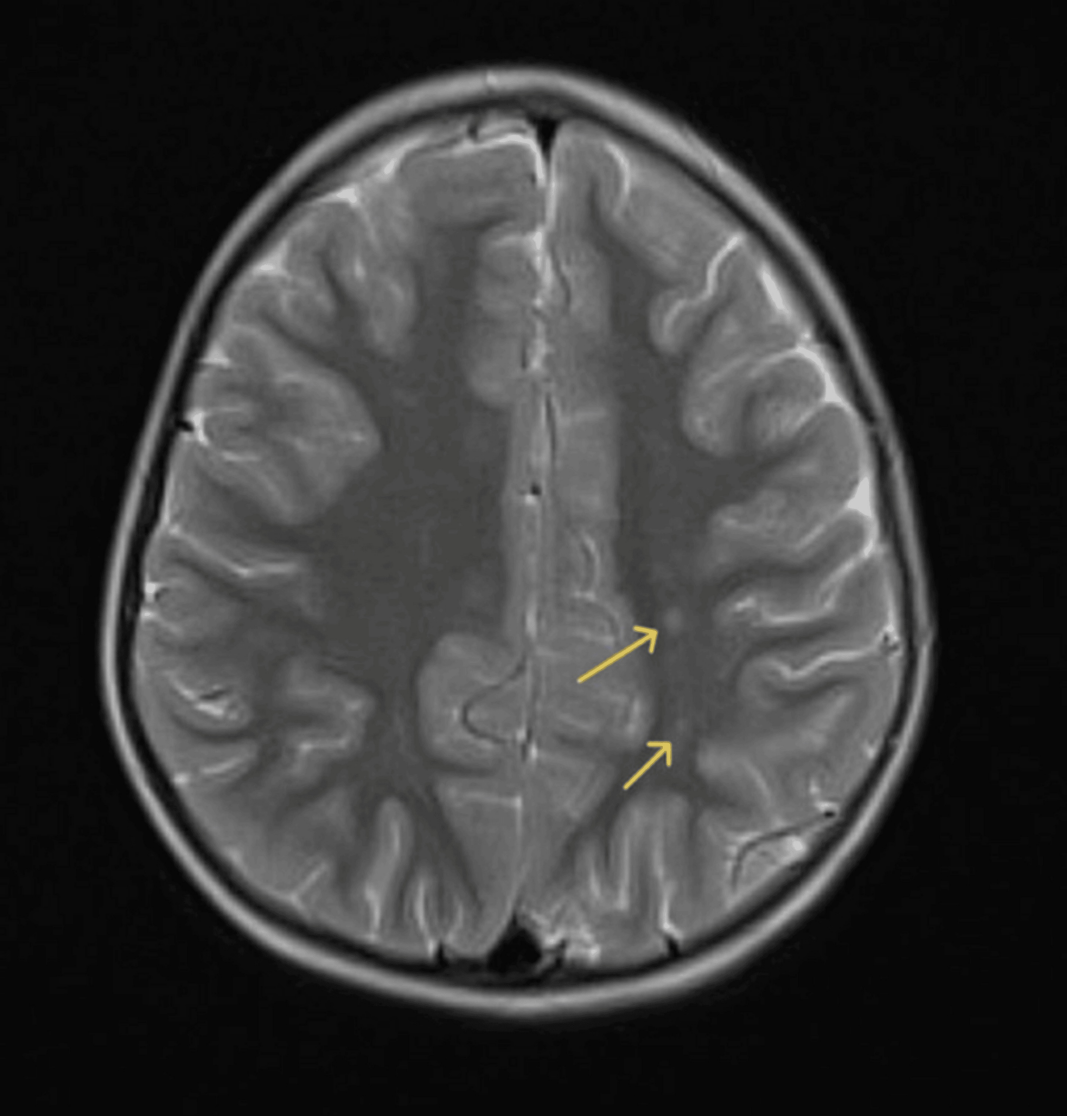
Follow-up MRI scan Follow-up axial T2-weighted MRI showing stable parasagittal dilated perivascular spaces in bilateral parietal white matter (arrows), without interval progression.

## Discussion

Chorea as a presenting symptom in a child should always raise suspicion of RF. In our case, although the child was far below the expected age threshold, we did not exclude the possibility of a rheumatic etiology, which proved to be correct.

Chorea often occurs weeks or even months after pharyngitis, making it difficult to establish a causal relationship. However, the presence of anti-streptolysin antibodies indicates the significance of this marker in raising suspicion of the disease.

The suspicion of RF prompted a more detailed cardiac evaluation of our patient. By utilizing the revised Jones criteria [5] and the introduction of serial echocardiography, even in patients without a clinical presentation of carditis, we were able to identify two major criteria and establish the diagnosis. In the initial echocardiogram, the minimal echocardiographic criteria for RHD defined by the latest World Heart Federation (WHF) guidelines [6] were not met, but in subsequent examinations, quantification of mitral and aortic regurgitation confirmed the diagnosis.

Cases of RF with chorea as presenting symptom are most often accompanied by cardiac involvement, either clinical or subclinical, in up to 70% of cases [7,8].

By definition, the term carditis in RF should encompass involvement of the pericardium, myocardium, and endocardium. However, as in our case, the most frequent manifestation of carditis in RHD is valvulitis, primarily affecting the mitral and aortic valves. Mitral regurgitation is present in approximately 60% of cardiac involvement in most studies [7,8]. Pericarditis and cardiac decompensation are rarely described.

The natural history of RHD is heterogeneous, showing potential for regression, stabilization, or progression. According to the latest WHF classification, our patient was categorized as Stage B, indicating a higher risk for disease progression. Given the patient’s age of less than five years, the likelihood of an unfavorable outcome was greater [9,10].

Despite this, we observed regression in the severity of mitral and aortic regurgitation within two months, continuing throughout the first year.

Current WHO positions based on meta-analyses and RCTs do not confirm that corticosteroid use affects the development or progression of RHD, but they do demonstrate an effect on chorea. According to a meta-analysis [11], the use of antibiotics, sodium valproate, and corticosteroids was associated with shorter chorea duration and a lower relapse rate. Patients receiving corticosteroid therapy for one month or longer had a monophasic disease course and shorter symptom duration. The corticosteroid regimen we used was shorter; however, the regression of chorea and mitral/aortic regurgitation was evident, and no relapses were documented in the first year following the initial episode. Considering the limitations of available studies [11,12], immunomodulatory therapy should become a focus of further research in RHD.

Currently, the only proven factor influencing disease progression is recurrence of RF [4]. Relapses most commonly occur within the first year following the initial episode. Recurrent RF may induce progressive or new valvular lesions detectable on echocardiography [13].

In general, recommendations for secondary antibiotic prophylaxis have remained unchanged over the years and are mostly based on national guidelines or on WHO recommendations where such guidelines are unavailable.

Adherence to secondary antibiotic prophylaxis remains a challenge. Long-term prophylaxis is difficult to maintain in low socioeconomic settings, but even in high-income countries, increasing drug shortages often force physicians to use alternative therapies. Oral penicillin formulations cannot maintain adequate serum drug concentrations for effective prophylaxis, resulting in suboptimal efficacy. In our case, this very young patient, classified as Stage B with a risk of progression, would require decades-long prophylaxis, feasible in principle but uncertain in terms of long-term parental and patient compliance.

In countries where RF remains endemic, the clinical importance of both primary and secondary prophylaxis is clearly evident, as the first manifestations of RHD are often heart failure and the need for surgical intervention [14]. In pediatric patients, mitral valve repair is preferred due to the potential complications of valve replacement, whereas in young adults, mitral valve replacement is more common [15]. The long-term outcomes of these procedures are generally favorable [16], but their invasive nature underscores the importance of early recognition and prevention of disease progression. Immunomodulatory therapy still holds promise in halting disease advancement.

## Conclusions

This case demonstrates that the burden of RF and RHD remains significant even in countries with relatively favorable socioeconomic conditions. The global increase in prevalence has largely resulted from improved access to echocardiography and the detection of subclinical carditis. However, without awareness of this disease and clinical suspicion, timely diagnosis will be missed. Immunomodulatory and immunosuppressive therapies offer hope for halting disease progression in diagnosed cases.
